# Effects of physical training on the immune system in diabetic rats

**DOI:** 10.4103/0973-3930.60010

**Published:** 2010

**Authors:** Daniel Maciel Crespilho, José Alexandre Curiacos de Almeida Leme, Maria Alice Rostom de Mello, Eliete Luciano

**Affiliations:** Department of Physical Education, São Paulo State University (UNESP), Rio Claro-SP, Brazil

**Keywords:** Physical training, immune system, metabolism and diabetes mellitus

## Abstract

**Aims:**

This study aims to investigate the influence of physical training on the immune system of diabetic rats.

**Materials and Methods:**

Adult male *Wistar* rats were distributed into Sedentary Control (SC), Trained Control (TC), Sedentary Diabetic (SD) and Trained Diabetic (TD) groups were used. Diabetes was induced by alloxan (32 mg/bw-i.v.). Training protocol consisted of swimming, at 32 ± 1°C, one hour/day, five days/week, supporting an overload equivalent to 5% of the body weight, during four weeks. At the end of the experiment the rats were sacrificed by decapitation and blood samples were collected for glucose, insulin, albumin, hematocrit determinations, total and differential leukocyte counting. Additionally, liver samples for glycogen analyses were obtained.

**Results:**

The results were analyzed by one way at a significance level of 5%. Diabetes reduced blood insulin, liver glycogen stores and increased blood glucose and neutrophil count. Physical training restored glycemia, liver glycogen levels, neutrophils and lymphocytes count in diabetic rats.

**Conclusions:**

In summary, physical training was able to improve metabolic and immunological aspects in the experimental diabetic rats.

## Introduction

Diabetes mellitus is a group of metabolic diseases characterized by hyperglycemia resulting from defects in insulin secretion, insulin action or both.[1] In muscle and fat cells, the uptake of circulating glucose depends on the insulin-stimulated translocation of the glucose transporter (GLUT) to the cell surface.[2,3]

The chronic insulinopenia and hyperglycemia of diabetes is associated with damage, dysfunction, and failure of various organs.[1] Regular exercise improves metabolic control in diabetic individuals and is an important component of treatment in diabetes mellitus. Exercise increases cardiorespiratory fitness, vigor, improves glycemic control, decreases insulin resistance, improves lipid profile, attenuates weight loss and enhances immune response.[4–7]

On the other hand, studies reported that intense and/or exhaustive training, long lasting acute exercises and insufficient terms of recuperation, lead to impairment of immune response. The decrease of serum glutamine has been cited as a factor that reduces the functionality of leucocytes increasing the susceptibility of athletes to infections. Skeletal muscles are the main source of serum glutamine, which is indispensable for leucocytes metabolism and functionality. Intensive exercise may increase the rate of consumption of this amino acid in the muscle causing a decrease of immune function.[8–9]

Sugiura and collaborators[8] studied the influence of chronic exercise training on immune system in voluntary running exercised rats. The protocol of voluntary running training was three days/week for eight weeks. After the exercise program the rats showed improvement in macrophage and lymphocyte functions.

Some studies show that blood lymphocyte number appears to be relatively unaffected in athletes that present overtraining and overreaching symptoms. These studies have pointed transient decreases in lymphocyte number during the initial stages (two to four weeks) of intensified training. It was found that cell counts normalized by the end of intensified training periods (4-8 weeks). Neutrophils appear to be the most affected leucocytes type on prolonged periods of intense exercise training, and it has been speculated that down regulation of neutrophil reflects an adaptive response to chronic inflammation resulting from microtrauma by daily intense exercise.[9–11]

On the other hand, little is known about the role of moderate exercise on immune system of diabetic organisms. Therefore, in this study we investigated the effects of physical training on metabolism and leucocytes count in experimental diabetic rats.

## Materials and Methods

Male Wistar rats were used in the experiments (180-210 g; 40-day-old). They were kept at 25°C with a light/dark cycle of 12 hour/12 hour, and fed with Purina rat food and water ad libitum. All experiments with the animals were performed in accordance with the specific Brazilian resolutions of the Bioethics of Experiments with animals (law N° 6.638 of May 8^th^ 1979; Decree N° 24.645 of July 10, 1934, Brazilian College of Animal Experimentation).

### Diabetes induction

Diabetes was induced by an intravenous injection (32 mg/kg b.w.) of Alloxan (Sigma). After five days, blood samples were obtained with animals in the fed state to determine the plasma glucose concentration. Rats which were not diabetic (<14,7 mmol/L) or too severely diabetic (>35,5 mmol/L) were eliminated from the study.

### Training protocol

For the study, the rats were randomly distributed in four groups (n = 8 per group), Sedentary Control (SC), Trained Control (TC), Sedentary Diabetic (SD) and Trained Diabetic (TD). The training included daily swimming with load of 5% of the body weight, one hour/day, five days/week, for four weeks.

### Analyses

Hematocrit was measured at rest, to ensure that measurements of metabolite and hormone concentrations were not influenced by changes in plasma volume. A capillary tube sample of blood was spun at 3,000 revolutions/minute for three minutes and the hematocrit was determined. Then, the rats were sacrificed 48 hour after their last exercise bout and blood samples were collected for glucose, insulin, and albumin determinations, and differential and total leucocytes counting. Samples of liver were used to evaluate glycogen contents.

Serum glucose concentration was measured by a colorimetric method.[12] Serum insulin concentration was determined by radioimmunoassay (RIA, Kit Coat-A-Count, USA). Colorimetric methods were employed for the measurements of glycogen concentrations.[13]

Total leucocytes count was performed using a specific pipette and a Neubauer blood- counting chamber. For differential leucocytes counting, the leucocytes were stained with panoptic dye and observed on Zeiss microscope.[14]

### Statistical analysis

All dependent variables were analyzed by one-way analysis of variance (ANOVA) and a significance level of *P* < 0.05 was used for all comparisons. The Bonferoni test was used for post-hoc comparisons. All results are expressed as mean ± SD.

## Results

After the experimental period, alloxan-induced diabetes decreased serum insulin and liver glycogen. Diabetes also increased glycemia, whereas physical training recovered liver glycogen and decreased glycemia [Table 1].

**Table 1:** Serum glucose (mg/dL), serum insulin (ng/ mL) and liver glycogen (mg/100 mg) after four weeks of experimental period

Group	Glucose	Insulin	Liver glycogen
SC	147.04 ± 9.96	1.56 ± 0.58	5.01 ± 0.98
TC	150.40 ± 13.07	1.35 ± 0.93	6.49 ± 1.14
SD	459.01 ± 185.77*,†	0.28 ± 0.19*,†	2.54 ± 1.67*,†
TD	269.4 ± 103,2‡	0.54 ± 0.16*,†	4.41 ± 1.60†

ANOVA; *P* < 0.05,

* CS;

† CT;

‡ DS. The values are the mean ± SD of 8 rats/group. SC - Sedentary Control; TC - Trained Control; SD - Sedentary Diabetic; TD - Trained Diabetic

Hematocrit and total leucocytes showed no differences among the experimental groups studied [Table 2]. Table 3 shows that, in differential leucocytes, diabetes increased neutrophils. Neutrophils counts were recovered by physical training.

**Table 2:** Hematocrit (%) and total leucocytes (×10^3^)after four weeks of experimental period

Group	Hematocrit	Total leucocytes
SC	52.0 ± 2.91	9.47 ± 2.93
TC	49.6 ± 2.3	9.25 ± 2.91
SD	52.4 ± 5.03	10.36 ± 3.57
TD	51.0 ± 2.91	8.26 ± 3.47

ANOVA; *P* < 0.05. The values are the mean ± SD of 5 rats/group. SC - Sedentary Control; TC - Trained Control; SD - Sedentary Diabetic; TD - Trained Diabetic)

**Table 3:** Neutrophils (%), eosinophils (%), lymphocytes (%) monocytes (%) after four weeks of experimental period

Group	Neutrophils	Eosinophils	Lymphocytes	Monocytes
SC	23.4 ± 3.2	6.0 ± 2.54	61.6 ± 5.59	9.0 ± 1.87
TC	27.2 ± 2.04	6.4 ± 2.3	57.4 ± 5.68	9.0 ± 2.34
SD	33.4 ± 7.09*	6.4 ± 1.81	53.6 ± 7.19	8.8 ± 2.04
TD	30.4 ± 1.14	3.4 ± 0.54	57.6 ± 2.5	9.6 ± 2.3

ANOVA; *P* < 0.05,

* CS. The values are the mean ± SD of 5 rats/group. SC - Sedentary control; TC - Trained control; SD - Sedentary diabetic; TD - Trained diabetic)

## Discussion

The effects of exercise in diabetic organism are of great interest to scientific community. The beneficial effects of physical activity on metabolic aspects are well known. Nevertheless, these effects on immune parameters remain uncertain.

As expected, alloxan induced-diabetes resulted in decreased blood insulin in both sedentary and trained rats. Diabetic animals showed hyperglycemia and the physical training decreased their blood glucose. Exercise and insulin stimulate glucose utilization synergistically and chronic exercise induces a decrease in blood glucose in experimental diabetes.[15–17] Several mechanisms may act locally to improve glucose uptake and disposal after exercise. These include increased muscle blood flow, increased insulin binding to its receptor (the insulin receptor, IR), increased IR turnover and increased glucose transport by stimulating GLUT4 translocation to the muscle cell surface.[15,18] Luciano *et al*.[19] showed that the increased responsiveness to insulin induced by chronic exercise in rat skeletal muscle may result, at least in part, from the modulation of the insulin signaling pathway at different molecular levels.

The present study showed no differences in hematocrit among the groups, indicating that the alterations observed in blood parameters were not influenced by dehydration.[20] The study demonstrates that diabetes decreased liver glycogen content and the physical training counteracted this alteration. Many studies have demonstrated that liver glycogen is important to maintain physical performance during prolonged exercise.[21,22] Exercise induces chronic adaptations in the liver, enabling diabetic rats to restore their glycogen stores.[22–23] The effects of insulin lead to a decrease in phosphorylase activity and an increase in glycogen synthase activity over that caused by glucose.[24]

The exercise-induced adaptations observed in the present study are in agreement with other studies which showed that trained rats had a higher total hepatic glycogen synthetase activity a lower phosphorylase activity and increased glycogen contents in liver.[25] These alterations in liver glycogen corroborate with previous studies.[26–27]

The effect of diabetes on the immune system has been the subject of much research. Some studies have verified increased in the susceptibility of persons with diabetes to complicated soft-tissue, urinary tract, and lower respiratory tract.[28–29] However, only a few studies attempted to elucidate the relationships among moderate physical training, diabetes mellitus and immune system.

Moderate exercise can decrease leucocyte adherence in blood vessels decreasing the incidence of chronic vascular diseases.[29] Leucocyte release during moderate endurance exercise is mainly related to demargination of cells adhering to the endothelium, this effect is caused by adrenergic stimulation and hemodynamic factors.[29] However, McFarlin *et al*.[30] analyzed the affects of repeated bouts of moderate exercise in the same day on circulating leukocyte count. Total leucocyte count was increased after a bout of exercise and remained so after two hours. Nevertheless, after 24 hours, leucocyte count returned turned to basal levels. In the present study, sacrifice occurred 48 hours after the last exercise session, explaining the return to basal levels of leucocyte count.

A number of important changes in the expression of neutrophils adhesion have been described during and following exercise classified in terms of the type, duration and intensity of the physical activity performed. Low intensities of endurance exercise do not affect either neutrophil adherence or the expression of adhesion molecules on neutrophils.[31] Perhaps the physical training protocol in our study did not interfere in neutrophil count because we utilized moderate intensity.

In our study, diabetes increased neutrophil count and physical training reversed the same, thus agreeing with studies that showed improvements in active subjects.[32–34] Neutrophils of diabetic patients do not increase their bactericidal activity in response to the same intensity of infection as compared to nondiabetic patients.[28] Diabetes decreases the affinity of the neutrophils with the endothelial cells. This effect, on the other hand, protects the lungs from neutrophils migration, thus decreasing its oxidative production. The increase in blood neutrophils observed might be a result of minor adherence of cell in target tissues. Physical training was able to reverse this parameter as well and may have generated other positive adaptations to organism.

The increase in cardiac output resulted from adrenalin activity release which causes eosinophils movement and discharge from lung, spleen and liver. The reversal of this effect takes 24 hours. It is probable that we found no differences in response to exercise in the present study because the rats were sacrificed after 48 hours of the last training session.[33,34]

Eosinophils are also altered in stress conditions. In the present study, the groups did not show interference from diabetes or physical training, indicating that the exercise swimming protocol here employed did not cause overtraining, since an increase in this parameter indicates overtraining.[35]

Some studies show an increase in the concentration of lymphocytes following exercise due to the recruitment of lymphocytes NK, cells T and B from the periphery of the body.[36] The present study showed that neither diabetes nor exercise caused alteration in the lymphocyte concentration.

Elevation of inflammation markers is frequently reported in diabetic organisms with increase in subpopulations of monocytes, which may contribute to the development of atherosclerosis.[37] On the other hand, the exercise usually produced a decrease in some subpopulations of monocyte count.[38] Nevertheless, in our study neither diabetes nor exercise caused alteration in the monocyte percentage.

## Conclusion

Our study showed that experimental diabetes induces metabolic damages and alters, at least in part, the differential leucocytes count. Also, moderate physical training was able to reverse the metabolic and immune parameters in diabetic rats. Future studies are required to evaluate the extent of the exercise influence in other experimental diabetes models.
